# Thrombocytopenia: A Diagnostic Dilemma and Incidental Detection of Systemic Lupus Erythematosus in a Middle-Aged Asian Male

**DOI:** 10.7759/cureus.10375

**Published:** 2020-09-11

**Authors:** Somnath Maitra, Sasmit Roy, Aveek Mukherjee, Srikanth Naramala, Subhasish Bose

**Affiliations:** 1 General Medicine, Jagannath Gupta Institute of Medical Sciences and Hospital, Kolkata, IND; 2 Nephrology, University of Virginia, Charlottesville, USA; 3 Nephrology, Liberty University College of Osteopathic Medicine, Lynchburg, USA; 4 Internal Medicine, Rutgers Robert Wood Johnson Medical School, New Brunswick, USA; 5 Rheumatology, Adventist Medical Center, Hanford, USA; 6 Nephrology/Internal Medicine, Lynchburg General Hospital, Lynchburg, USA

**Keywords:** systemic lupus erythematosus, thrombocytopenia, male lupus, complement levels, anti-double-stranded dna

## Abstract

Systemic lupus erythematosus is a multisystem disorder much more common in females than males due to the effect of the hormone estrogen. There are also specific differences in clinical presentation in men and women. We present a unique case of a 54-year-old middle-aged Asian male presenting with only generalized weakness without other systemic features and with only incidental finding of thrombocytopenia. Notable laboratory values were positive for antinuclear antibody (ANA) and anti-double-stranded DNA (dsDNA), low complement 3 level with normal complement 4 levels, along with severe thrombocytopenia and mild anemia. The patient was eventually diagnosed with systemic lupus erythematosus based on these parameters. Bone marrow biopsy revealed an increased number of megakaryocytes without hypocellular or hypercellular marrow and no dysplasia of cell lines. He was initiated on oral prednisone, and his symptoms recovered remarkably with normalization of lab values upon discharge. The case's importance lies in the fact that the diagnosis of lupus can be missed in male patients with nonspecific clinical features due to certain differences in presentation from females. This diagnosis should be included in the workup of any thrombocytopenia.

## Introduction

Systemic lupus erythematosus (SLE) is a connective tissue disorder with various modes of presentation. Often, the clinical features are nonspecific and pose challenges in diagnosis. The case discussed here presented with nonspecific symptoms along with incidental thrombocytopenia in laboratory findings. Age and male sex posed a diagnostic challenge. There are subtle differences in the presentation of lupus disease in males and females, which clinicians should be mindful of. SLE is associated with hematological complications, such as hemolytic anemia, leukopenia, and thrombocytopenia. Thrombocytopenia is mediated by antiplatelet autoantibodies or rarely associated with azathioprine, methotrexate, and rarely hydroxychloroquine (i.e., the various drugs used to treat lupus disease). Sometimes, thrombocytopenia is severe (<20,000 to 50,000/mm^3 ^) in 3%-10% of patients posing risk of devastating bleeding effects [1,2]. Treatment of thrombocytopenia in lupus disease is primarily done with immunosuppressive therapy, which includes corticosteroids and other agents such as mycophenolate mofetil, azathioprine, rituximab, and thrombopoietin receptor agonists like eltrombopag and romiplostim. Treatment with intravenous immunoglobulin (IVIG), although very effective in primary immune thrombocytopenia, has not been shown to offer long-term benefit in secondary immune thrombocytopenia like lupus disease [3]. Fostamatinib, a recently developed spleen tyrosine kinase inhibitor, has shown promises in therapy of chronic immune thrombocytopenia [4]. The primary concern with thrombocytopenia is prevention of the hemorrhagic complications.

## Case presentation

 A 54-year-middle-aged Asian male patient, a clinical pharmacist by occupation, presented to the emergency department with a generalized weakness for the last three months without any history of night sweats, chills and rigors, weight loss, loss of appetite, vomiting, diarrhea, abdominal pain, joint pain, rashes, and urinary symptoms. There was no history of hemoptysis, hematemesis, or melena. The patient had no medical comorbidities nor had any history of illicit drug abuse or exposure to radiation or chemicals. He did not have any documented evidence of receiving heparin within the last few months. He was not on any prescription medications and neither taking any over-the-counter supplements. He was a nonsmoker, nonalcoholic, and had no family history of lupus or other autoimmune disorders.

Physical examination was grossly unremarkable with normal vital signs, i.e., temp 98^o^F, blood pressure 128/64 mm Hg, heart rate 78 beats/min, and pulse oximetry of 97% room air. There was mild pallor without icterus, no cyanosis, or clubbing. There was mild pitting edema in bilateral lower extremities with equal peripheral pulses bilaterally. There were two to three oral soft palate petechial spots with no other detectable skin lesions. Systemic examination, including integumentary, gastrointestinal, cardiovascular, and musculoskeletal, was unremarkable. There was no restriction of joint movements. Laboratory values are shown in Table 1.

**Table 1:** Investigations from day 1 of admission GFR: glomerular filtration rate; AST: aspartate aminotransferase; ALT: alanine aminotransferase; ANA: antinuclear antibody; HEP2: human epithelial cell type 2; CCP: cyclic citrullinated peptide; DNA: deoxyribonucleic acid; ESR: erythrocyte sedimentation rate; gm: gram; mg: milligram; mEq: milliequivalent; DL: deciliter; L: liter; mm: millimeter; min: minute; hr: hour; IU: international unit

Parameters	Normal values	Day 1	Day 3	Day 5	Day 7	Day 9	Day 12
Hemoglobin	13.5-17.5 gm/dL	10.2 gm/dL			10.3 gm/dL		
Total leukocyte count	4,000-11,000/mm^3^	4,200/mm^3^			4,400/mm^3^		
Platelet count	150,000-400,000/mm^3^	40,000/mm^3^	26,000/mm^3^	58,000/mm^3^	84,000/mm^3^	110,000/mm^3^	140,000/mm^3^
Fasting plasma glucose	<100 mg/dL	90 mg/dL					
Blood urea nitrogen	7-20 mg/dL	32 mg/dL					
Creatinine (GFR)	0.8-1.2 mg/dL (90-120 mL/min)	1.2 mg/dL (78 mL/min)					
Total bilirubin	<1.2 mg/dL	1.0 mg/dL					
AST	7-40 U/L	39 U/L					
ALT	7-40 U/L	42 U/L					
Alkaline phosphatase	88-126 U/L	120 U/L					
Albumin	3.5-5 gm/dL	3.0 gm/dL					
Globulin	2-3.5 gm/dL	3.2 gm/dL					
Sodium	135-145 mEq/L	135 mEq/L			137 mEq/L		136 mEq/L
Potassium	3.5-5.1 mEq/L	4.2 mEq/L			4.0 mEq/L		3.9 mEq/L
Rheumatoid factor			Negative				
Anti-CCP antibody			Negative				
ANA HEP2	<1: 80		Positive >1:80 (homogenous)				
Anti-double-stranded DNA (Crithidia)	<20 IU/mL		Positive (160 IU/mL)				
Anti-Smith antibody			Negative				
Anti-phospholipid antibody			Negative				
Complement 3	0.9-1.8 gm/L		0.8 gm/L				
Complement 4	0.16-0.48 gm/L		0.2 gm/L				
ESR	0-22 mm/hr		74 mm/hr				
C-reactive protein	<10 mg/L		56 mg/L				
24-hr urinary protein	0-150 mg/24 hr		900 mg/24 hr				

A provisional diagnosis of immunogenic thrombocytopenic purpura was made with the differential diagnosis of hypoplastic anemia and myelodysplastic syndrome.

**Figure 1:**
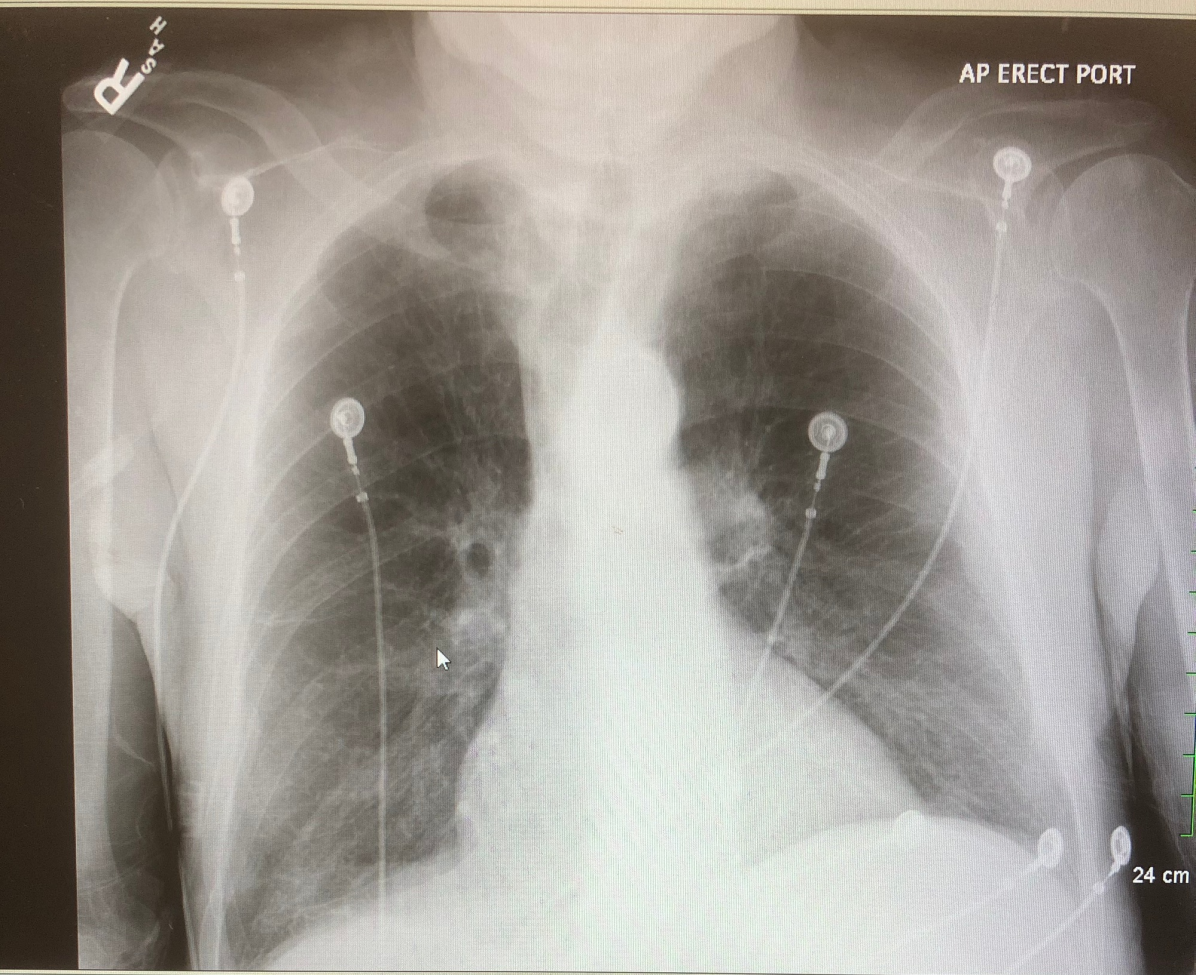
Normal Chest X-Ray

The investigations revealed no abnormality on bleeding time, clotting time, prothrombin time, activated partial thromboplastin time, and international normalized ratio (INR). Hepatitis viral serology and HIV were nonreactive. Peripheral blood smear showed only normocytic normochromic anemia with no abnormal cells. Serum levels of vitamin B12 and folate were normal. As shown in Table 1, complement 3 level was decreased, while erythrocyte sedimentation rate (ESR) and C-reactive protein (CRP) were elevated. Chest X-ray (Figure 1), ultrasound abdomen, electrocardiogram, and echocardiogram were all normal. A bone marrow examination was conducted that showed only a mild increased number of megakaryocytes.

A diagnosis of SLE was made according to the 2019 European League Against Rheumatism/American College of Rheumatology classification criteria for SLE (score ≥10) based on thrombocytopenia, raised 24-hour urinary protein, positive anti-double-stranded DNA (dsDNA) and low complement 3 levels [5]. The patient was subsequently started on oral prednisone 50 mg daily. Platelet counts started to rise after oral steroids initiation on day 4 of admission and reached to near normal levels upon discharge. The patient was discharged on day 13 after admission with a follow-up appointment at Rheumatology Clinic. His hospital course was prolonged due to social issues of proper disposition. He is regularly being followed in their clinic with normal platelet counts at present and no lupus flares. His steroids have been tapered off, and currently, he is on oral hydroxychloroquine/Plaquenil®. 

## Discussion

SLE is a multisystem disorder affecting females far more than males [6]. The effect of estrogen is supported by certain observations that include the female to male ratio among various age groups. In children, the female to male ratio is 3:1, but in adults, it is 7-15:1. Older persons have an 8:1 ratio. In a study by Budhathoki et al., the ratio was as high as 10:1 [7]. Male patients of SLE have similar kidney disease features, dermatological disease characteristics, joint manifestations as females, with less of photosensitivity and more of serositis, older age of presentation, and higher mortality at one year [8].

Lymphopenia, thrombocytopenia, low complement 3 levels, and anti-dsDNA positivity are more common in men. Myocardial infarction is more common in men due to risk factors like hypertension, but dermatological manifestations are less common. A decreased incidence of Raynaud’s phenomenon has been found in three previous studies [9-11]. The difference in gender is not only due to hormones. In animal models like mice, polymorphism of the Y chromosome, inactivation of the X chromosome, X chromosomal gene dosage, and parental imprint may all affect autoimmunity [12-17].

Thrombocytopenia is an important hematological complication of SLE [18]. The pathogenesis is multifactorial, but the commonest mechanism is increased clearance of platelets by antiplatelet autoantibodies. Other mechanisms are thrombotic thrombocytopenic purpura, disseminated intravascular coagulation, hemophagocytic syndrome, anti-phospholipid syndrome, and impaired thrombopoiesis. Thrombocytopenia is reported in 20%-40% of patients of SLE. It may be the initial feature of lupus in 16% of patients, presenting months or maybe 10 years before diagnosis. Many studies have found that thrombocytopenia is associated with higher mortality, although the onset and severity have not been clearly defined [19]. Treatment options are similar to idiopathic thrombocytopenic purpura, i.e. with intravenous glucocorticoids, rituximab, and the newer emerging therapies of thrombopoietin agonists like eltrombopag and romiplostim. The last option reserved is splenectomy in these refractory cases. As mentioned earlier, although IVIG is an established treatment option in primary immune thrombocytopenia, its role in secondary thrombocytopenia has not been well established. In the study by Arnal et al., there was rapid response in platelet count in matter of few days but no long-term benefits were observed [3]. The newly approved fostamatinib, a novel spleen tyrosine kinase inhibitor, has shown great promise in therapy of refractory immune thrombocytopenia [4]. This was approved recently in USA by FDA in April 2018, and more studies of its effectiveness in secondary causes are required.

## Conclusions

The case's importance lies in the fact that SLE is a diagnosis that is often missed in males due to its rare association. The presentation is different to some extent in males than females, as mentioned above. In the diagnostic workup of thrombocytopenia in men, SLE must be considered as a differential, so that timely management can be instituted. Thrombocytopenia may lead to an acute presentation in SLE with life-threatening circumstances. Emergency therapy in the forms mentioned above is often required to prevent hemorrhagic complications, and a complete or partial platelet response is needed for recovery. Maintenance therapy is thereby required for the prevention of further relapses. Generally, patients with platelet counts more than 50,000/mm^3^ without any bleeding manifestations do not require treatment unless they have a history of hemostatic disorders, anticoagulant therapy, surgery, or trauma. In our case, as the patient presented with severe thrombocytopenia, corticosteroids were required as the initial therapy to prevent catastrophic hemorrhagic complications.
